# Spineless cactus cladode is a viable replacement to barley and maize grains in the feed rations of dromedary camel calves

**DOI:** 10.1002/vms3.1227

**Published:** 2023-07-25

**Authors:** Ashraf Alkhtib, Muhannad Muna, Mohammad Darag, Iyad Alkhalid, Ziad Al‐asa'ad, Hanaa Mfeshi, Roba Zayod, Emily Burton

**Affiliations:** ^1^ Nottingham Trent University, School of Animal Rural and Environmental Sciences, Brackenhurst Campus Southwell Nottinghamshire UK; ^2^ General Commission of Scientific Agricultural Research (GCSAR) Damascus Syria

**Keywords:** Camelid, feed conversion ratio, growth

## Abstract

**Background:**

No studies determined the use of spineless cactus cladodes in camel diets.

**Objectives:**

The effect of replacing the main energy source in camel diets with fresh spineless cactus cladodes on growth performance was determined. Furthermore, the ability of morphology to predict cladodes yield was determined.

**Methods:**

A prediction model of spineless cactus cladode weight based on cladode volume was developed. Three cladodes per plants were randomly selected from 100 plants. Weight and volume were then recorded for each cladode. Sixteen male camel calves (196 ± 18.2 kg live weight and 430 ± 5.55 days of age) were allotted to treatments, control (conventional camel fattening diet based on cotton seed hulls, cereal grains and agro‐industrial by‐products) or cactus (barley and maize grains in the control diet were replaced totally by fresh cactus cladode on dry matter basis). The study contained a 100‐days growth trial and a 21‐day digestibility trial (15 days of adaptation and 7 days of faeces collection). Blood samples were collected monthly from each animal.

**Results:**

Cactus cladode volume predicted the dry weight with a high accuracy (prediction error = 3.5%). Nutrient intake and nutrient digestibility did not significantly differ among the dietary treatments (*p* > 0.05). The treatment significantly decreased feed conversion ratio by 1.52 points (*p* < 0.05). All blood parameters were within the normal range of dromedary camels.

**Conclusions:**

Spineless cactus cladode is a potential replacement to the conventional energy sources in dromedary camel diets.

## Graphical abstract

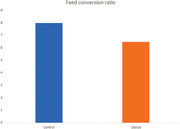

## 1 INTRODUCTION

Camels have an increasingly important role in the livelihood of humans in some geographical regions. The overall population of camels around the world is recently reported as 37,509,000 heads with 27,361,000 heads are kept by the farmers in the arid and semi‐arid areas of the developing countries (FAOSTAT, 2019). Previously, camel production was practised within nomadic communities. However, they are now an important component of modern communities in arid regions (Faye, 2016).

The dromedary camel is an excellent source of meat particularly in dry areas where climate negatively impacts the performance of other livestock. This is due to physiological features which enable the dromedary camel to tolerate high temperature, solar radiation, water scarcity and poor nutrition. Camels have relatively low growth rate (500 g/day) (Ali et al., 2019).

To date, grains and grains by‐products have been the main energy source of ruminant in the dry areas of the developing countries. However, the decrease in cereal yields as a result of severe drought and global climate change resulted in an increase in the price of cereal grains and their by‐products (Ben Salem & Smith, 2008). Therefore, the use of cheaper and more sustainable alternatives is now on the top of priorities of agricultural sectors in developing countries.

Spineless cactus (*Opuntia ficus‐indica*) is a multipurpose plant which is grown by farmers for food production (fruits) and livestock feeding (cladodes). Moreover, it is used to fence farms and homes (Alary et al., 2007). Generally, spineless cactus yields high amount of dry matter ranging from 3.1 to 47.3 t/ha depending on biotic and abiotic factors (Dubeux et al., 2006). Spineless cactus cladode contains high levels of non‐structural carbohydrates and calcium (16.5–52.3 g/kg) (Heuzé & Tran, 2017). It is highly variable in crude protein (2.6%–11.4%) and neutral detergent fibre (21.4%–37.7%) (Heuzé & Tran, 2017). Supplementation of straw‐based diets with spineless cactus cladodes has been shown to enhance nutrient digestibility of sheep (Ben Salem et al., 1996). However, in addition to these favourable attributes, high levels of oxalates have been found in spineless cactus cladodes. Oxalates at a level of 1.1 g/kg live weight in sheep diets have been reported to cause failure in kidney functions, urolithiasis, hypocalcemia and a performance reduction (D'Mello, 1997). More recently, however, it has been established that feeding spineless cactus in combination with fibre‐rich feeds tends to alleviate the potential negative consequences associated with oxalates (Ben Salem et al., 2002a).

Determining cactus cladodes yield using a non‐destructive method is essential for livestock feeding planning in the farm. Plant morphology has high potential to be used to determine cladode yield in cactus since it showed strong association with forage yield in *Atriplex* (Ben Salem et al., 2005) and Reed Canarygrass (Casler & Hovin, 1985).

To our knowledge, there is no published data on the effect of spineless cactus cladode on dromedary camel growth performance and health. Furthermore, the ability of morphology to predict cladode yield of spinless cactus is not studied. Therefore, the primary objective of this study is to determine the effect of replacing cereal grains by spineless cactus cladodes on growth performance, nutrient digestibility and health of dromedary camels. In addition to that, the current study aims at determining a simple and robust equation to predict cactus cladodes yield using simple morphology measurements.

## 2 MATERIALS AND METHODS

### 2.1 Camels, treatments and housing

Sixteen growing dromedary male camels (live weight of 209 ± 5.2 kg and age of 430 ±5.54 days) were randomly and equally allotted into two dietary treatment groups. All the experimental camels belonged to Shami breed (endogenous Syrian breed). The animals were kept in individual pens in open‐sided shed for the whole of the trial.

The control group was fed a standard camel ration (5.66 g/kg live weight barley grain + 2.18 g/kg live weight maize grain + 2.61 g/kg live weight cotton seed meal + 3.63 g/kg live weight wheat bran + 9.5 g/kg live weight cotton seed hulls). In other words, the control diet consisted of 24% barley grain, 9.25% maize grain, 11.05% cotton seed meal, 15.4% wheat bran and 40.3% cotton seed hulls. The fresh spineless cactus cladode replaced barley and maize grain on dry matter basis in the cactus group. All the experimental animals were stall‐fed without grazing. Camels were steeped with Ivermectin (200 mcg/kg live weight) to control common parasites, vaccinated against common diseases of camels (anthrax, pasteurellosis and enterotoxaemia) and then adapted to the experimental pens and the dietary treatments for 2 weeks before the 120‐day fattening trial.

Spineless cactus cladodes were obtained daily from 4‐year‐old plants grown in an experimental plot within the research station. The animals were kept in individual pens in an open‐sided barn and fed two times/day two equal portions (at 09:00 and 16:00 h). Clean water and salt licks were available all the time to the experimental camels. The remaining concentrate and roughage feeds were collected prior to morning feeding and weighed on daily basis for every camel then stored at −20°C for feed analyses. Live weight of the experimental camels was measured before morning feeding at day 1 of the trial and every 10 days to obtain weight gain and feed conversion ratio.

### 2.2 Nutrient digestibility

At the last day of the fattening trial, all animals were fitted with faeces collection bags and went through a collection period of 7 days after 14 days of adaptation to faeces bags. Faeces of each camel was collected on daily basis, weighed and subsampled at a rate of 10%. Faces subsamples were oven‐dried at 55°C to reach a constant weight, ground to pass through 1 mm sieve and stored for further analyses. The digestibility (Apparent digestibility of protein) of the experimental diets was calculated from data of the nutrient intakes and the losses in faeces.

### 2.3 Blood sampling and analysis

Blood sampling was done at the start of the trail then monthly. Two blood samples were obtained from each camel through the jugular vein in a heparin tube and an in non‐heparin tubes prior to the morning diet. The non‐heparin blood tubes were centrifuged (1677 × *g*; 20 min; 4°C) then the sera samples were stored at −20°C for further analyses. Albumin, total protein, glucose, cholesterol, urea, packed cell volume, haemoglobin, alanine transferase, aspartate transferase, creatinine and glutamate oxaloacetate transaminase were determined using specific commercial kits (Katal) and a UV spectrophotometer at the recommended wavelengths. Haemoglobin and packed cell volume were determined using an automated haematology analyser (Diatron, Abacus 5).

### 2.4 Feed and faeces analyses

The feed and faeces samples were dried at 105°C overnight in a forced air oven to determine the dry matter content (Association of official analytical chemists [AOAC], 2003; method 934.01). Ash content was determined using a muffle furnace at 550°C (AOAC, 2003; method 942.05). Kjeldahl (AOAC, 2003; method 954.01) and Soxhlet method (AOAC, 2003; method 920.39) were used to analyse nitrogen and ether extract, respectively. Nitrogen content of samples was multiplied by 6.26 to obtain crude protein content. Neutral detergent fibre was analysed as per recommended by Van Soest et al. (1991) without the use of an alpha amylase. Neutral detergent fibre in this study was presented as residual ash exclusive. Total and soluble oxalates were analysed according to Moir (1953). Total tannins were determined according to Makkar (2003).

### 2.5 Spineless cactus cladodes biomass and morphology data

A total of 100 spineless cactus plants were randomly selected to represent the experimental field and three cladodes were randomly selected from each plant. The longest and shortest diameter, thickness and sundry weight were recorded for each cladode. The cladode surface area (cm^2^) was calculated as follow: the long diameter (cm) × the short diameter (cm) × *π*. Cladode volume (cm^3^) was calculated by multiplying the surface area by the thickness.

### 2.6 Statistical analysis

Blood data and growth performance data were analysed separately. A repeated measurements design was used to analyse blood data with the following specification:

$$Y_{ij}=\;\mu +TRT_{\left(i\right)}+M_{\left(j\right)}+Animal_{\left(k\right)}+{\left(TRT\times M\right)}_{ij}+\varepsilon_{ijk}$$

where *Y* is the response variable, *TRT* is the effect of the treatment, *M* is the effect of the measurement, *Animal* is the effect of the camel, *TRT* × *M* is the effect of the interaction between treatment and measurement and *Ɛ* is the residual.

The effect of the treatment on nutrients digestibility and growth performance was analysed according to the following model:

$$Y_{ij}=\;\mu +TRT_{i}+\varepsilon_{ij}$$

where *Y* is the response variable, *TRT* is the effect of the treatment and *Ɛ* is the residual. Fisher's least significant difference at level of 0.05 was used for means separation in both models.

The cladode measurement data was divided into two sets, a calibration set and a validation set using Puchwein (1988) algorithm. The algorithm identified 24 cladodes for the blind validation. Three models were constructed using the calibration set then blindly validated using the validation set. R software version 3.6.1 was used to analysis the study data (R Core Team, 2017).

## 3 RESULTS

### 3.1 Spineless cactus cladode biomass prediction

**FIGURE 1:**
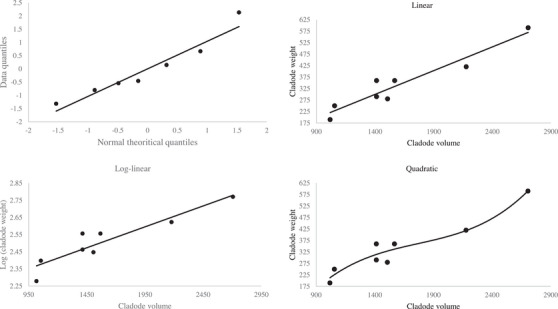
Normal Q–Q plot of cactus cladode weight (g) and its relationship with cladode volume (cm^3^).

Figure 1 shows that the distribution of spineless cactus cladodes weight and the relation between cactus cladode weight and volume. The correlation between the cladode weight and morphometry was strong (*r* > 0.7; *p* < 0.001) (Table 1). Table 2 presents the performance of prediction models of cactus cladodes weight using cladodes morphology. The three models had similar coefficient of determination (0.854–0.911). Yet, the log‐linear model had the lowest 95% percentile prediction error.

**TABLE 1:** Linear correlation between cactus cladode weight and morphology.

	*r*	*p* Value	Min–max
Cladode length	0.856	***	26–39 cm
Cladode width	0.771	***	12–17 cm
Cladode thickness	0.81	***	8–13 cm
Cladode area	0.911	***	1017–2081 cm^2^
Cladode volume	0.995	***	1017–2706 cm^3^
Cladode weight	–	–	10–59 g

*Note*: ‘*’, ‘**’, ‘***’: significant at 0.05, 0.01 and 0.001, respectively.

**TABLE 2:** Performance of models predicting cladode weight using cladode volume.

Model	Simple linear^a^	Log 10 simple linear^b^	Quadratic^c^
Coefficient of determination	0.91	0.854	0.911
Residual standard error	34.7	0.054	34.7
95% percentile calibration error	16.9	3.97	19.2
95% percentile prediction error	16.2	3.5	17.7

^a^ Simple linear = *Cladode weight* (g) = 1.16 + 0.0206 × *volume* (cm^3^).

^b^ Log‐linear = $Cladode\;weight\;\left(g\right)=10^{\left(0.2121+volume\left({\mathrm{cm}}^{3}\right)\times 2.431\times 10^{-5}\right)}.$

^c^ Quadratic model = $Cladode\;weight\;\left(g\right)=\;5.5+volume\left({\mathrm{cm}}^{3}\right)\times 0.0154+volume{\left({\mathrm{cm}}^{3}\right)}^{2}\times 1.4\times 10^{-6}.$

### 3.2 Growth performance and blood metabolites

The nutritional composition of the experimental diets is presented in Table 3. Table 4 shows the daily intake of dry matter, organic matter, crude protein as well as the nutrient digestibility of the experimental camels. The experimental treatment did not significantly affect the daily intake of dry matter, organic matter and crude protein of the experimental camels (*p* > 0.05). The digestibility of dry matter, organic matter, crude protein, neutral detergent fibre, nitrogen free extract and ether extract of cactus group was not significantly different from the control group (*p* > 0.05).

**TABLE 3:** Nutritional composition (g/kg dry matter) of the experimental feedstuffs.

Feed ingredient	Barley grain	Maize grain	Cotton seed meal	Wheat bran	Spineless cactus cladodes	Cotton seed hulls	Control diet	Cactus diet
Dry matter	900	890	920	890	10	900	90	60.5
Organic matter	970	985	928	949	825	917	94.2	89.3
Crude protein	112	89	380	151	61	42.2	11.7	10.3
Ether extract	20	44	110	40	22	8.1	3.04	2.9
Neutral detergent fibre	217	122	337	450	260	771	48.1	50.4
Total oxalate					143		0	4.75
Soluble oxalate					58.4		0	1.94
Total tannins					12.8		0	0.426
% in the diet								
Control diet	24	9.25	11.05	15.4	0	40.3		
Cactus diet	0	0	11.05	15.4	33.25	40.3		

**TABLE 4:** Effect of replacing barley and maize grains by spineless cactus cladodes on nutrient intake and digestibility of dromedary camels.

Item	Control_a_	Cactus_b_	SEM	*p*‐Value
*Intake (g/live weight0.75)*				
Dry matter	93.5	90.3	4.87	ns
Organic matter	83.5	79.1	5.56	ns
Crude protein	10.4	9.09	1.85	ns
*Digestibility coefficient (%)*				
Dry matter	73.2	77.1	6.65	ns
Crude protein	65.5	66.3	4.99	ns
Nitrogen free extract	72	67.9	5.32	ns
Neutral detergent fibre	54.3	57.1	5.43	ns
Ether extract	64.6	67.5	3.54	ns

*Note*: ‘*’, ‘**’, ‘***’: significant at 0.05, 0.01 and 0.001, respectively.

Abbreviation: SEM, standard error mean.

^a^ Control = 5.66 g/kg live weight barley grain + 2.18 g/kg live weight maize grain + 2.61 g/kg live weight cotton seed meal + 3.63 g/kg live weight wheat bran + 9.5 g/kg live weight cotton seed hulls.

^b^ Cactus = 2.61 g/kg live weight cotton seed meal + 3.63 g/kg live weight wheat bran + 9.5 g/kg live weight cotton seed hulls + 7.84 g/kg live weight fresh spineless cactus cladodes.

The feed consumption of the camels was not significantly affected by the treatment (*p* > 0.05) and nor was the final body weight. The camels fed the cactus diet had significantly (*p* ≤ 0.05) lower daily growth and feed conversion ratio (by 124 g/day and 1.52 points, respectively) compared to the control (Table 5).

**TABLE 5:** Effect of replacing barley and maize grains by spineless cactus cladodes on growth performance of dromedary camels.

Item	Control^a^	Cactus^b^	SEM^a^ ^,^	*p*‐Value
Initial live weight (kg)	210	208	7.04	ns
Final live weight (kg)	274	259	8.01	ns
Average daily gain (g/day)	638	514	20.8	**
Daily dry matter intake (g/day)	4360	4310	33.1	ns
Daily dry matter intake (g/kg^0.75^)	78.6	78.5	1.81	ns
Feed consumption (kg)	488	487	1.04	ns
Feed conversion ratio	8	6.48	0.315	**

*Note*: ‘*’, ‘**’, ‘***’: significant at 0.05, 0.01 and 0.001, respectively.

Abbreviation: SEM, standard error mean.

^a^ Control = 5.66 g/kg live weight barley grain + 2.18 g/kg live weight maize grain + 2.61 g/kg live weight cotton seed meal + 3.63 g/kg live weight wheat bran + 9.5 g/kg live weight cotton seed hulls.

^b^ Cactus = 2.61 g/kg live weight cotton seed meal + 3.63 g/kg live weight wheat bran + 9.5 g/kg live weight cotton seed hulls + 7.84 g/kg live weight fresh spineless cactus cladodes.

The measurement, animal and the treatment × measurement interaction did not significantly alter blood chemistry parameters of the camels (*p* > 0.05) (Table 6). The cactus‐based diet significantly decreased the albumin, total protein, urea, packed cell volume and haemoglobin of the experimental camels (*p* < 0.05). Glucose, cholesterol, alanine transferase, aspartate transferase, creatinine and glutamate oxaloacetate transaminase were not significantly affected by the treatment (*p* > 0.05).

**TABLE 6:** Effect of replacing barley and maize grains by spineless cactus cladodes on blood metabolites of dromedary camels.

	Treatment			*p*‐value^c^		
Item	Control^a^	Cactus^b^	SEM^c^	Treatment	*M*	*T* × *M*
Albumin (g/L)	37.8	34.8	0.536	***	ns	ns
Total protein (g/L)	55.3	52.1	0.756	***	ns	ns
Glucose (mg/dL)	129	128	3.21	ns	ns	ns
Cholesterol (mg/dL)	20.5	20.2	1.02	ns	ns	ns
Urea (mg/dL)	48.8	41.5	2.11	ns	ns	ns
Packed cell volume (%)	30.4	27.6	0.312	***	ns	ns
Haemoglobin (g/dL)	15.6	14.6	0.228	***	ns	ns
Alanine transferase (IU/L)	3.5	4	0.12	ns	ns	ns
Aspartate transferase (IU/L)	24.8	30.2	5	ns	ns	ns
Creatinine (mg/dL)	1.6	2.23	0.35	ns	ns	ns
Glutamate oxaloacetate transaminase (IU/L)	80.3	87.8	3.65	ns	ns	ns

*Note*: ‘*’, ‘**’, ‘***’: significant at 0.05, 0.01 and 0.001, respectively.

Abbreviation: SEM, standard error mean.

^a^ Control = 5.66 g/kg live weight barley grain + 2.18 g/kg live weight maize grain + 2.61 g/kg live weight cotton seed meal + 3.63 g/kg live weight wheat bran + 9.5 g/kg live weight cotton seed hulls.

^b^ Cactus = 2.61 g/kg live weight cotton seed meal + 3.63 g/kg live weight wheat bran + 9.5 g/kg live weight cotton seed hulls + 7.84 g/kg live weight fresh spineless cactus cladodes.

^c^ *T*: Effect of dietary treatment; *M*: effect of measurement; *T* × *M*: effect of treatment–measurement interaction.

## 4 DISCUSSION

### 4.1 Spineless cactus biomass prediction

A major goal of the current study was to develop a non‐destructive method to determine cactus yield of cladodes using cladode morphology. The distribution of cactus cladodes weight was close to normal with some deviation which suggests a log transformation of cladode weight before regressing. The log‐linear model had the lowest 95% percentile prediction error. Therefore, this model could be used to predict 95% of cactus cladodes weight using a combination of cladode length, width and thickness with an error less than 3.5%. The simple prediction model developed in the current study offers a non‐destructive, simple and accurate method to estimate spineless cactus cladodes biomass which would offer a viable method to help farmers to accurately determine the amount of cactus cladodes to be included in daily livestock diets. Future studies should continue to develop similar models to predict the nutritive value of cactus cladodes using morphological traits.

### 4.2 Growth performance and blood metabolites

The hypothesis of the current study is that spinless cactus cladodes could be used as the energy source in dromedary camel diets since it is rich in soluble carbohydrates.

The chemical composition of spineless cactus cladodes was within the range reported by Heuzé and Tran (2017). The results of the current study showed that both extract and neutral detergent fibre of spineless cactus cladodes were close to that of barley grains. However, these cladodes had less crude protein compared to barley grains. This is in agreement with Abidi et al. (2009) who also evaluated the nutritive value of cactus cladodes. The current study showed a high content of both total and soluble oxalate in spineless cactus cladodes. Ayadi et al. (2009), Ben Salem and Abidi (2009) and Stintzing and Carle (2005) documented the nutritional composition and anti‐nutritional factors of cactus cladodes. The high content of moisture and non‐structural (soluble) carbohydrates in spineless cactus cladodes suggests that there may be some risk of disturbing rumen fermentation (particularly altering rumen pH) causing acidosis in ruminants. However, the current study indicated that there was no significant difference in nutrients digestibility between cactus and control. This may be due to the protective mechanism induced by the mucilage secretions of spineless cactus cladodes; whereby the mucilage induces more salivation, thus preventing a pH decrease through the buffering effect of increased saliva in the rumen. This is in agreement with Ben Salem et al. (1996) and Tegegne et al. (2007) who also found no adverse effect of spineless cactus cladodes on rumen pH when fed to sheep. Additionally, the presence of pectin in the spineless cactus could be responsible for providing buffering power thus preventing pH reduction (Duskova & Marounek, 2001).

Spineless cactus cladodes have high content of oxalate which could cause damage in rumen wall and the kidney tubules (James, 1978). Yet, camels in both control and cactus groups did not show toxification symptoms and all blood metabolites were within the normal range of healthy camels. This is because the majority of the oxalates in cactus cladodes are insoluble, therefore, they precipitate as insoluble calcium in the rumen (Ben Salem et al., 2002b). Additionally, camels seem to show high oxalates tolerance compared to other livestock species (Dadvar et al., 2019).

In the current study, the digestibility and the organic matter intake and blood glucose were similar between the control and cactus group. This pinpoints that those camels fed the control and cactus diets received similar amount of energy. Blood albumin and total protein of the experimental camels were decreased due to the dietary treatment. This indicates to a reduction in protein supply in the small intestine. In the rumen, pectin is fermented at a faster rate and to greater extent compared to other carbohydrates (Marounek & Dušková, 1999). Thus, microbial protein synthesis seems to be reduced when feeds rich on pectin (like spinless cactus cladodes and pulps) replaced cereal grains ruminant diets (Balcells et al., 1992) due to the poor synchrony between the supply of energy from cactus and nitrogen from the concentrate. This, together with the lower protein content of cactus cladodes compared to maize and barley grains, could result in a reduction of amino acid flow into the duodenum that would negatively affect animal performance (Beever et al., 1988). This is in agreement with Richardson et al. (2003) and Castrillo et al. (2004) study based on lambs supplemented with barley vs. sugar beet pulp.

The decrease in feed conversion ratio resulted from the dietary treatment in the current study could relate to the increase in feed utilization efficiency or a change in carcass composition, although the latter was not determined in this study. Further studies should determine the effect of spineless cactus cladodes on carcass characteristics and meat quality of camels.

Dromedary camels are kept by the farmers in arid and semiarid areas where there is a limited access to natural pasture and cheap feed. Yet, integrating spineless cactus, as a source of food and cash in such areas would supply dromedary camels with a cheap energy comparable that of to the traditional energy sources like barley and maize grains. These expensive grains, replaced by spineless cactus cladodes, would be alternatively used for human consumption and poultry nutrition. That would improve the overall food security in arid and semiarid areas in the world.

In conclusion, the use of spineless cactus cladodes as energy source in total replacement of maize and barley grains of dromedary camels’ diets decreased the feed amount consumed for the production of 1 kg of live weight by 19%. Furthermore, no adverse effect on the health was found on the dromedary camels fed on spineless cactus cladodes in replacement of maize and barley grains. Accordingly, using spinless cactus cladodes as camel feed would decrease the cost of meat production and allow more grains to be used for human and poultry nutrition which would improve the food security of the people in dry areas.

Volume could be used to predict dry weight of spineless cactus cladode using a simple linear model. That model would offer a none‐destructive tool to estimate spineless cactus cladode yield.

## AUTHOR CONTRIBUTIONS

Mohammad Darag, Iyad Alkhalid, Ziad Al‐asa'ad, and Roba Zayod performed the trials. Ashraf Alkhtib did the statistical treatment. Muhannad Muna, Mohammad Darag, Iyad Alkhalid, Ziad Al‐asa'ad, Hanaa Mfeshi and Roba Zayod formulated the diets and advise on camels’ care and feeding. Hanaa Mfeshi analysed the samples Ashraf Alkhtib and Muhannad Muna wrote the draft. All authors provided advice, revised the progress of the manuscript and approved the final manuscript.

## CONFLICT OF INTEREST DISCLOSURE

The authors declare that they have no conflicts of interest.

## FUNDING STATEMENT

Not applicable.

## ETHICS STATEMENT

All study procedures were approved by the Ethical Committee of Damascus University, Syria (ECD1057/2022).

### PEER REVIEW

The peer review history for this article is available at https://publons.com/publon/10.1002/vms3.1227.

## Data Availability

Data of the current study is available from the corresponding author on reasonable request.
